# Supplementary material for: High discrimination ratio, broadband circularly polarized light photodetector using dielectric achiral nanostructures
Source: Light Sci Appl. 2024 Sep 27;13:275. doi: 10.1038/s41377-024-01634-8 (PMC11427471; doi:10.1038/s41377-024-01634-8)
Supplement: Supplementary file 1 — Supplementary Information for High Discrimination Ratio, Broadband Circularly Polarized Light Photodetector Using Dielectric Achiral Nanostructures [file 41377_2024_1634_MOESM1_ESM.docx]

**Supplementary Information for**

**High Discrimination Ratio, Broadband Circularly Polarized Light Photodetector Using Dielectric Achiral Nanostructures**

Guanyu Zhang^1^, Xiaying Lyu^1^, Yulu Qin^1^, Yaolong Li^1^, Zipu Fan^2^, Xianghan Meng^1^, Yuqing Cheng^3*^, Zini Cao^1^, Yixuan Xu^1^, Dong Sun^2^, Yunan Gao^1, 4, 5^, Qihuang Gong^1, 4, 5^, and Guowei Lyu^1, 4, 5*^

*^1^ State Key Laboratory for Mesoscopic Physics, Collaborative Innovation Center of Quantum Matter, Frontiers Science Center for Nano-Optoelectronics, School of Physics, Peking University, Beijing 100871, China*

*^2^ International Center for Quantum Materials, School of Physics, Peking University, Beijing 100871, China*

*^3^ School of Mathematics and Physics, University of Science and Technology Beijing, Beijing 100083, China*

*^4^ Collaborative Innovation Center of Extreme Optics, Shanxi University, Taiyuan, Shanxi 030006, China*

*^5^ Peking University Yangtze Delta Institute of Optoelectronics, Nantong, Jiangsu 226010, China*

** Corresponding author:* [*yuqingcheng@ustb.edu.cn*](mailto:yuqingcheng@ustb.edu.cn)*(Y.Cheng),* [*guowei.lyu@pku.edu.cn*](mailto:guowei.lyu@pku.edu.cn) *(G. Lyu)*


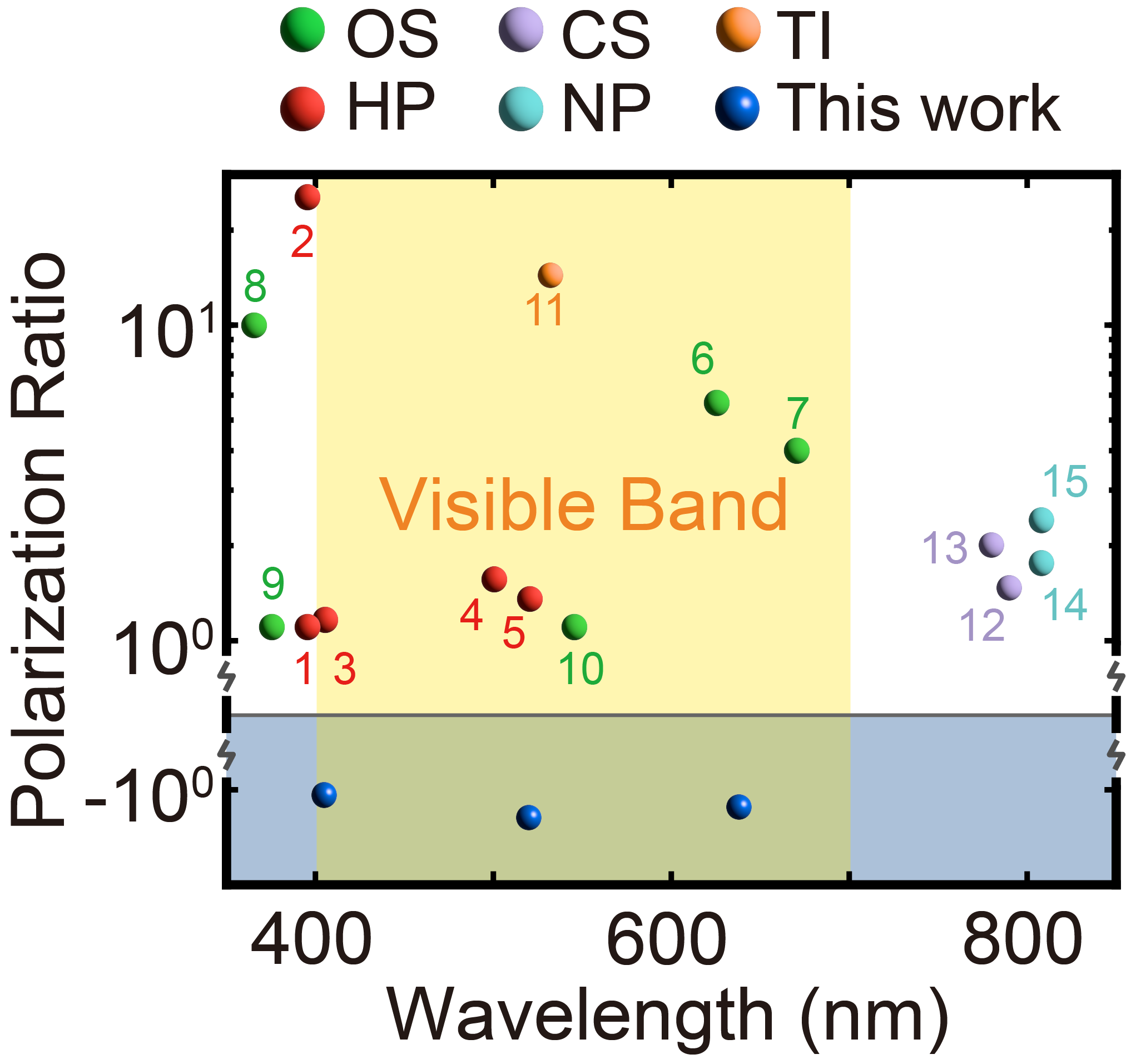


**Fig. S1** **Comparison of polarization ratio (PR).** Comparison of the achiral-structure-based device with other CPL photodetectors through PR^1-15^. The yellow area represents the visible band, while the blue area indicates the region where R_LCP_ and R_RCP_ reverse.


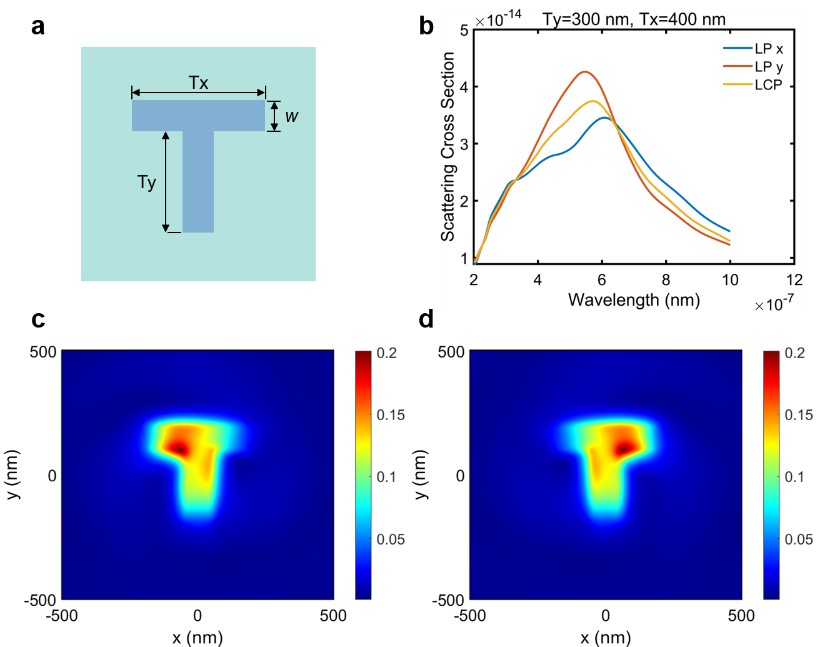


**Fig. S2 Chirality-resolved near field modes of T-shaped grooves. a.** Structure of the simulated devices. Here, Tx=400 nm, Ty=300 nm, w=100 nm. The thickness and the etching depth is 120 nm and 100 nm. **b.** Scattering cross section versus wavelength curves for different polarization states. **c, d.** Near-field modes excited by CPL of opposite chirality at 520 nm wavelength. The light fields are concentrated on different sides of the T-shaped groove, indicating a chiral-resolved near-field mode. The figure above illustrates that our CPL detection scheme is not limited to specific structures.


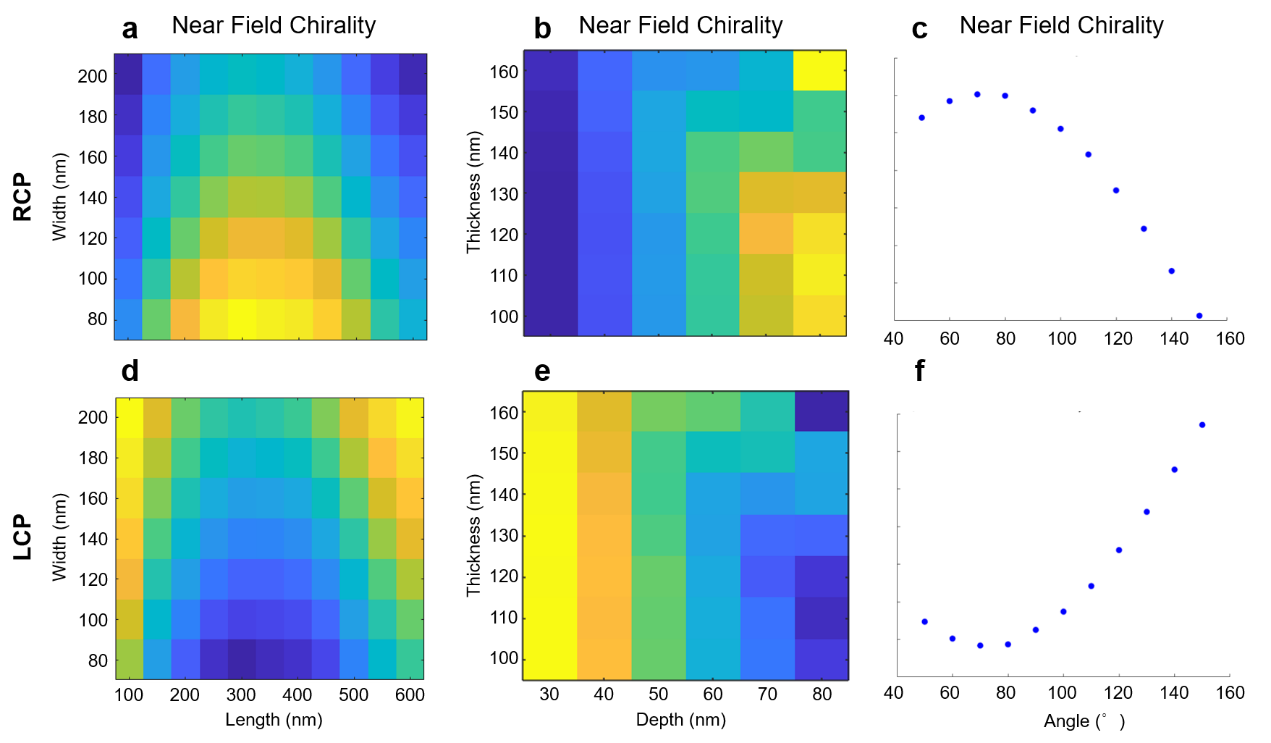


**Fig. S3 Geometry parameter optimization of the V-grooves. a, d.** Near-field chirality varies with arm length and arm width. **b, e.** Near-field chirality varies with the thickness of nanosheets and the depth of grooves. **c, f.** Near-field chirality varies V-shape angle. Fig. S2a-c show the near field chirality calculated under RCP light while Fig. S2d-f show the near field chirality calculated under LCP light.


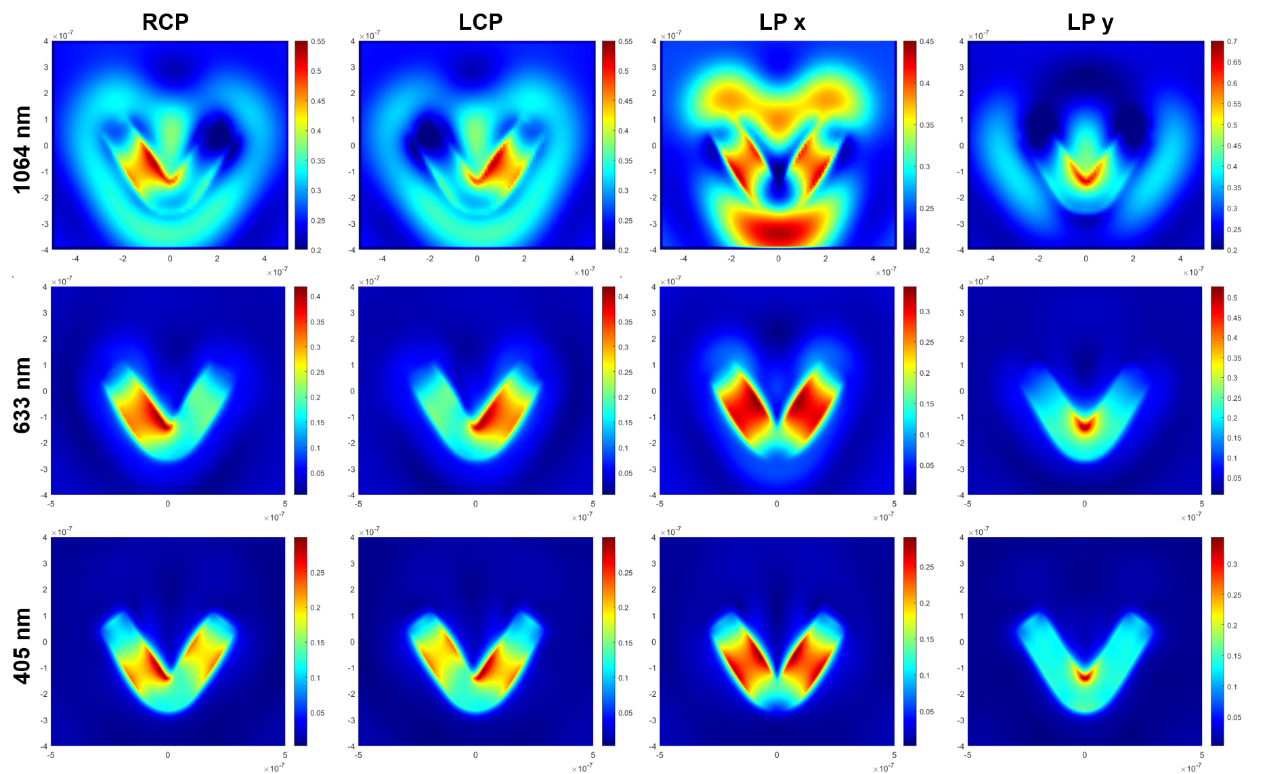


**Fig. S4 Near-field modes of V-groove under different wavelengths and polarization states.** The three rows from top to bottom sequentially show the near-field modes of the V-shaped groove under light illumination at 1064 nm, 633 nm, and 405 nm. The four columns of figures from left to right show the near-field modes excited by RCP, LCP, LP x and LP y light at different wavelengths. The geometric parameters of the V-groove are consistent: *l*=300nm; *w*=100nm; *θ*=70°.


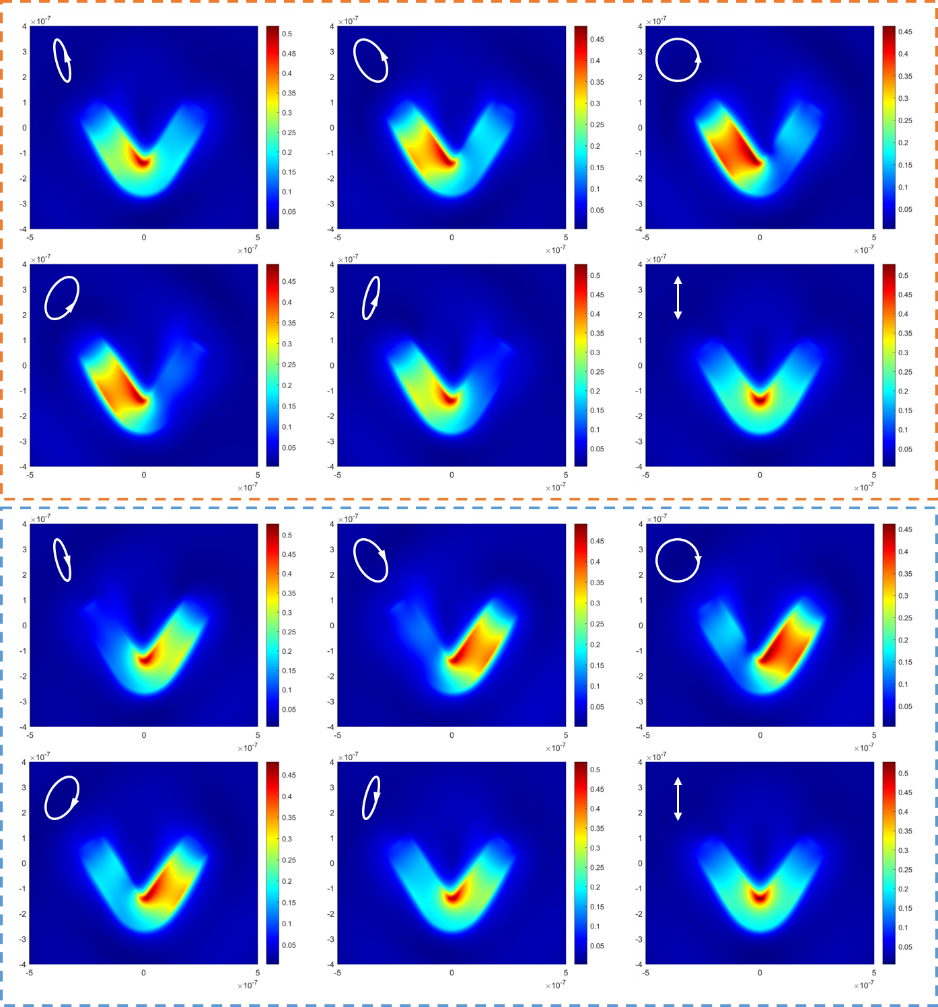


**Fig. S5 Near-field modes of V-grooves under different polarization states as the QWP rotates.** From left to right and from top to bottom, the electric field intensity near V-grooves under different polarization states are shown. Between two adjacent images, the rotation angle of the QWP differs by 15°. The corresponding polarization states are shown in the upper right corner. Excitation light of opposite chirality, indicated by dashed orange and blue frames, results in the concentration of near-field light on different sides of the V-groove.


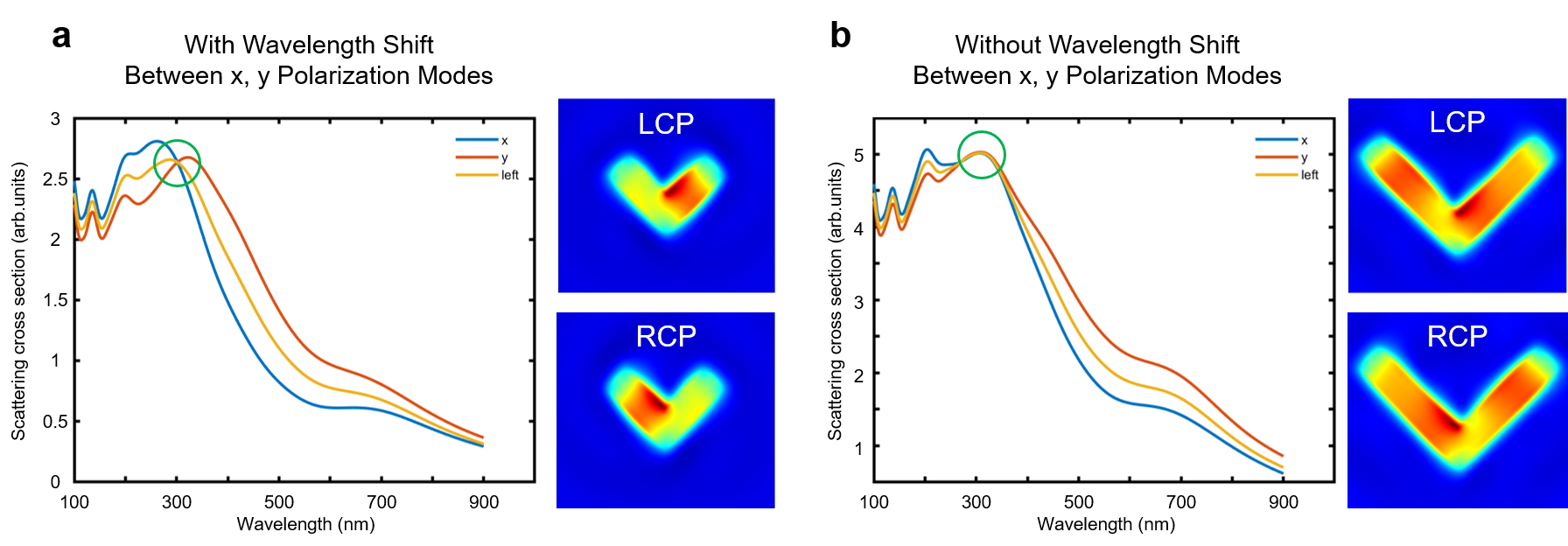


**Fig. S6 The relationship between the detuning of x, y polarization mode and the chiral-resolved near-field mode. a.** The V-groove (*l*=300 nm, *w*=100 nm, *θ*=90°) with resonance shift between LP x and LP y mode possess LCP-dependent chiral-resolved near-field mode. **b.** V-shaped grooves with longer arm lengths (*l*=500 nm, *w*=100 nm, *θ*=90°) exhibit little detuning in the resonance peaks for x and y polarizations, resulting in a weak chiral resolution of their near-field modes.


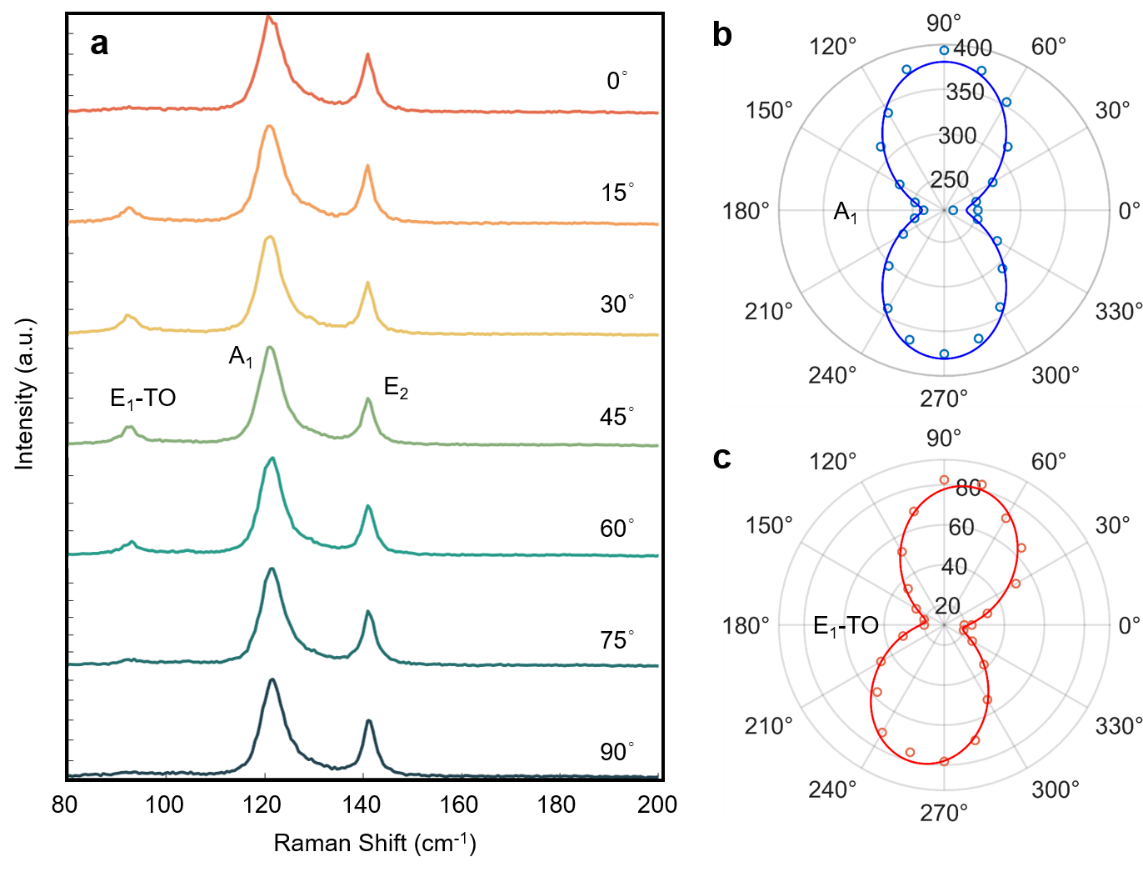


**Fig. S7 Polarization-dependent Raman spectroscopy of Te nanosheets. a.** Raman spectrum changes with the rotation angle of half-wave plate. **b.** The variation curve of the A1 Raman peak intensity with the polarization angle of the light field. The 0° direction corresponds to the orientation parallel to the long axis of the Te nanosheets. **c.** The variation curve of the E_1_-TO Raman peak intensity with the polarization angle of the light field.


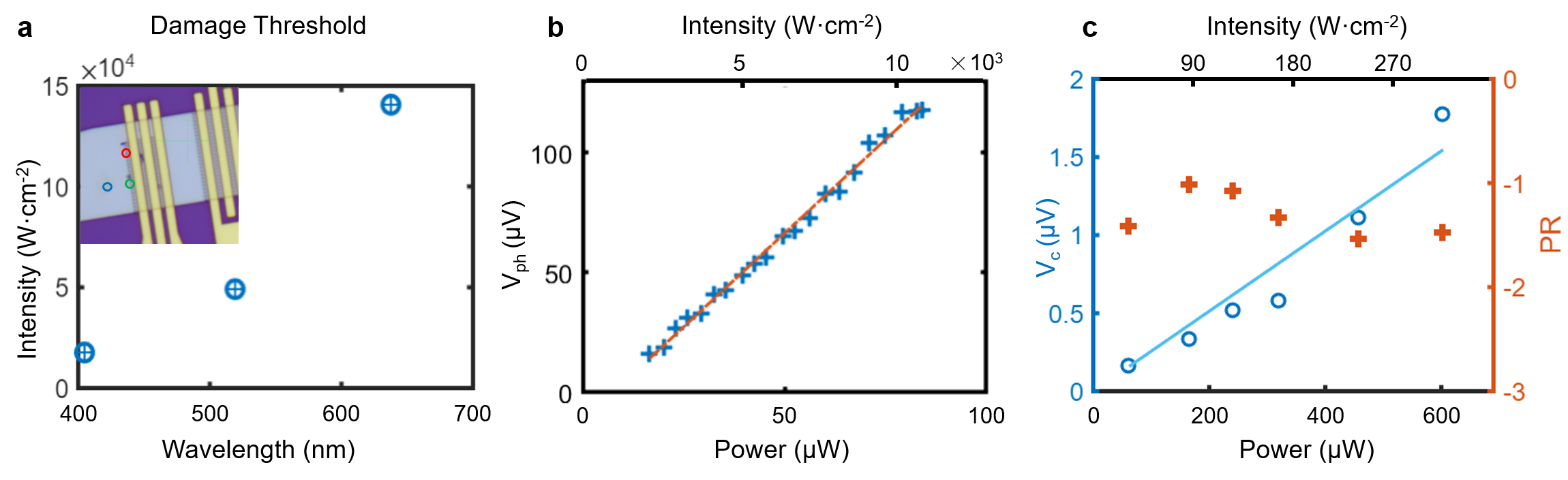


**Fig. S8 Device optical response varies with incident power. a.** Damage light intensity threshold of the device. This threshold was measured by irradiating the device with focused light spots of different wavelengths and intensities for 1 minute. The minimum light intensity that causes observable damage is recorded. The inset shows a microscope photo of the device used for testing, with circles indicating damage to the nanosheet caused by threshold-intensity lasers at different wavelengths. **b.** The photovoltage of a unit device varies with incident power under a 520 nm laser. **c.** The CPL-sensitive photovoltage and polarization ratio of an array device varies with incident power. The CPL discrimination capability remains stable across the tested power range.


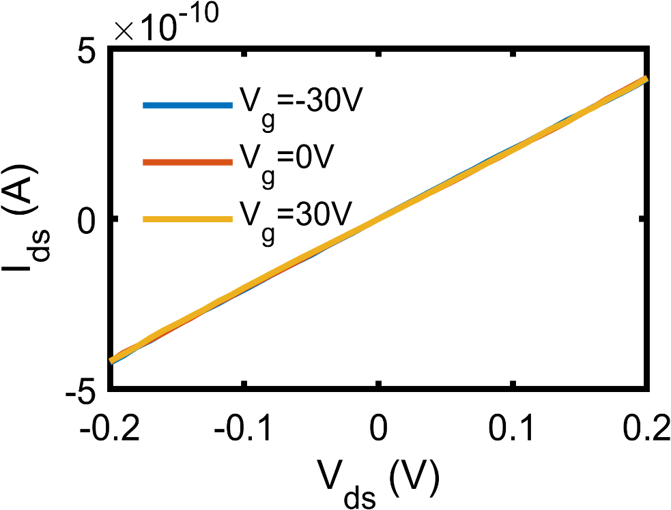


**Fig. S9 I-V curve. a.** The device leakage current varies linearly with the source-drain voltage, suggesting an ohmic contact between the electrodes and the Te nanosheets. Due to the thickness of the nanosheets, the source-drain current is minimally affected by the gate voltage.


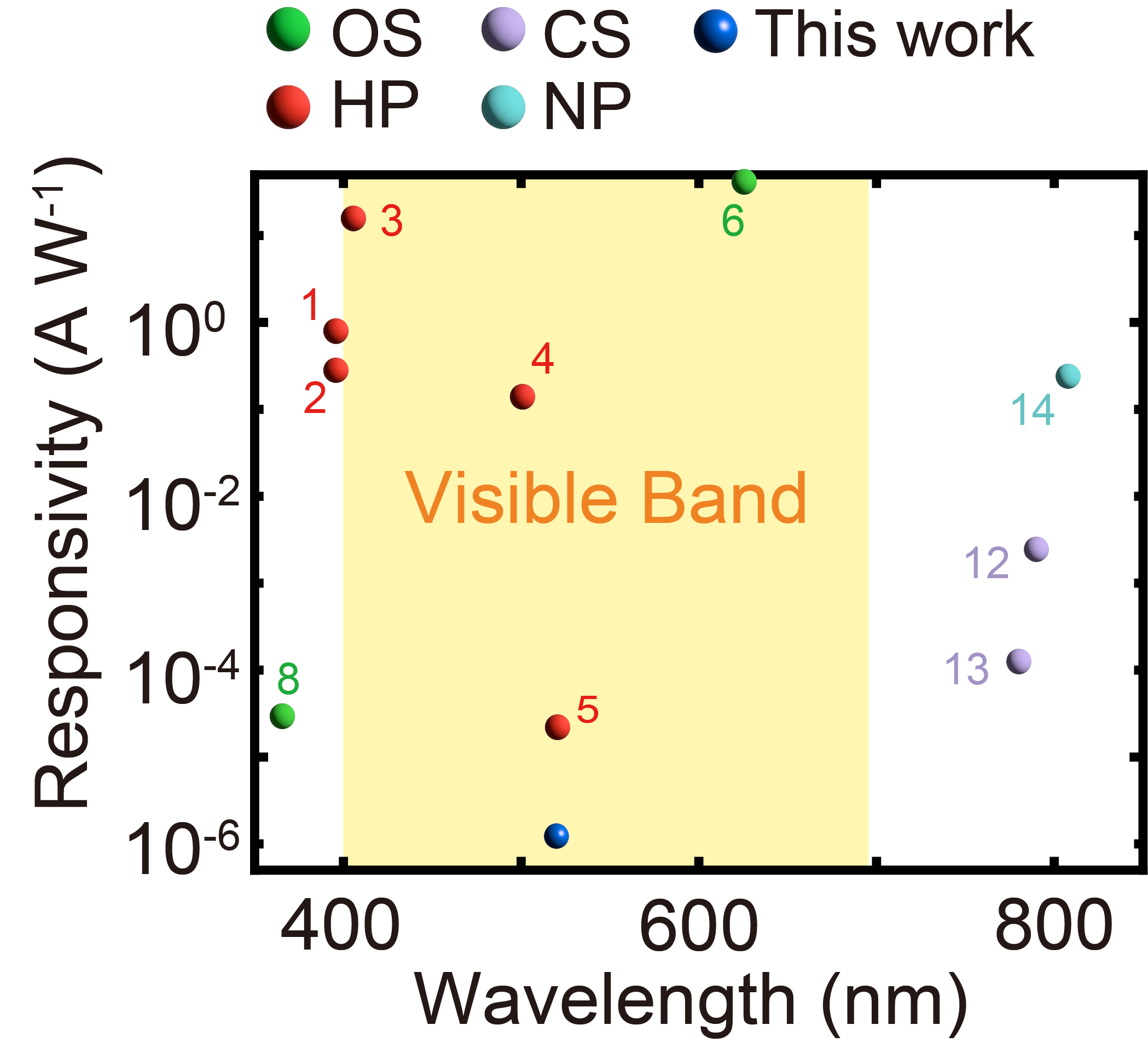


**Fig. S10 Comparison of** **photocurrent response.** Comparison of our achiral-structure-based device with other CPL photodetectors through responsivity. To make our device comparable with most devices operating based on photovoltaic or photoconductivity effects whose responsivities are in the unit of A W^-1^, we also measured the device's current response under CPL light excitation^1-5, 6, 8, 12-13^.


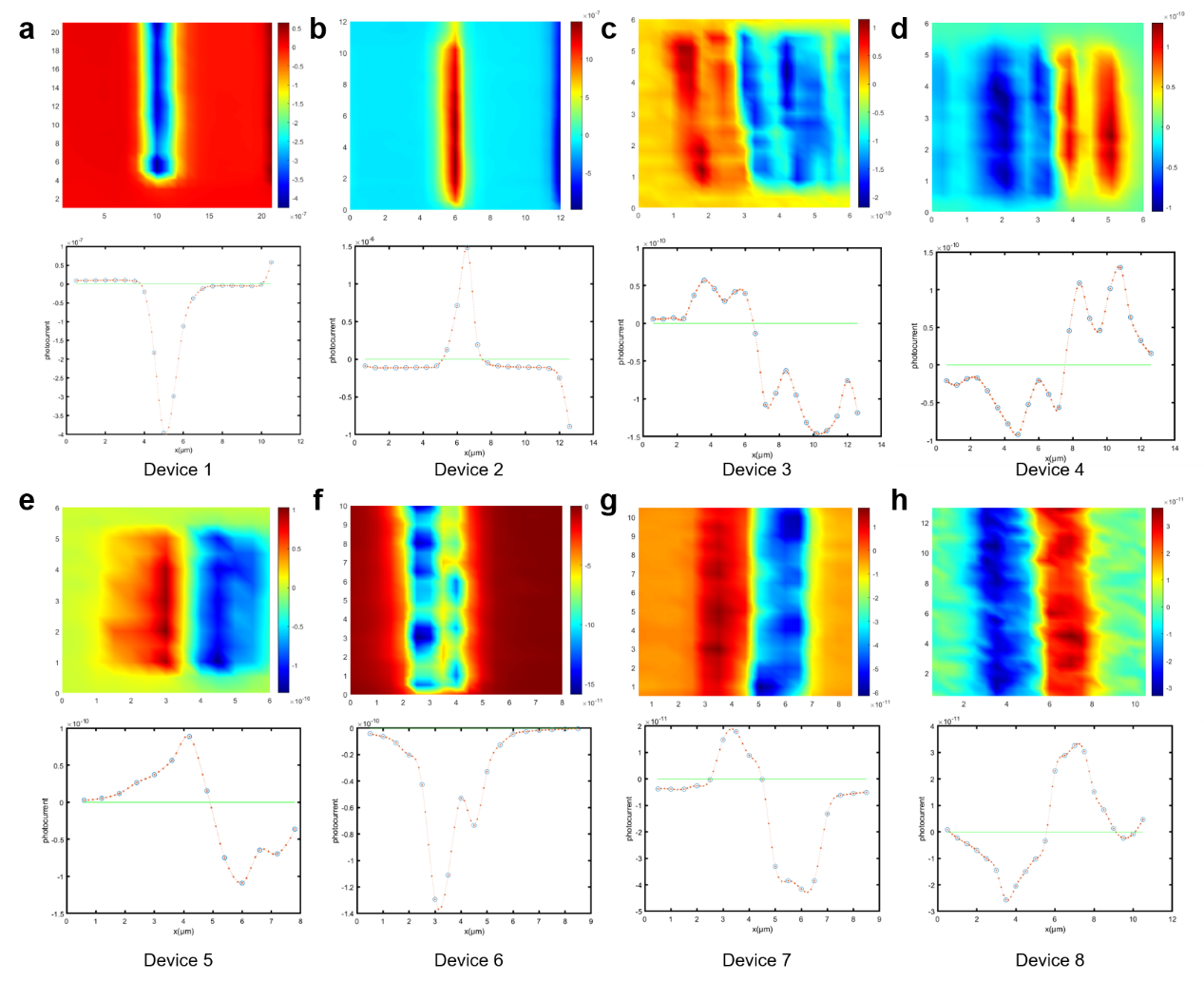


**Fig. S11 Photocurrent mapping of multiple devices. a, b.** Photocurrent mapping of two devices fabricated by Laser Direct Writing (Top) and the photocurrent on a transverse section line of the device (Bottom). The photocurrent neutral line in the bottom figure is marked with a green line. The electrodes on either side exhibit significant asymmetry, causing the photocurrent generated by the temperature rise in one electrode to completely overshadow the photocurrent produced by the other electrode. As a result, the photocurrent scanning image displays a single-peak form. **c-h.** Photocurrent mapping of six devices fabricated by electron beam lithography (Top) and the corresponding photocurrent on a transverse section line (Bottom). With improvements in fabrication accuracy, it is observed that electrodes on both sides generate photocurrents in opposite directions under focused light spot illumination. However, due to random variations in the electrodes’ contact properties, the magnitudes of the photocurrents on the two sides differ. This difference is entirely random, and situations have even arisen where only one electrode-nanosheet interface can generate a photocurrent, as show in Fig. S8f. In some mapping results, the photocurrent excited by a single electrode exhibits a double-peak shape (Fig. S8c, d), representing the responses generated at the inner and outer side of the electrode.


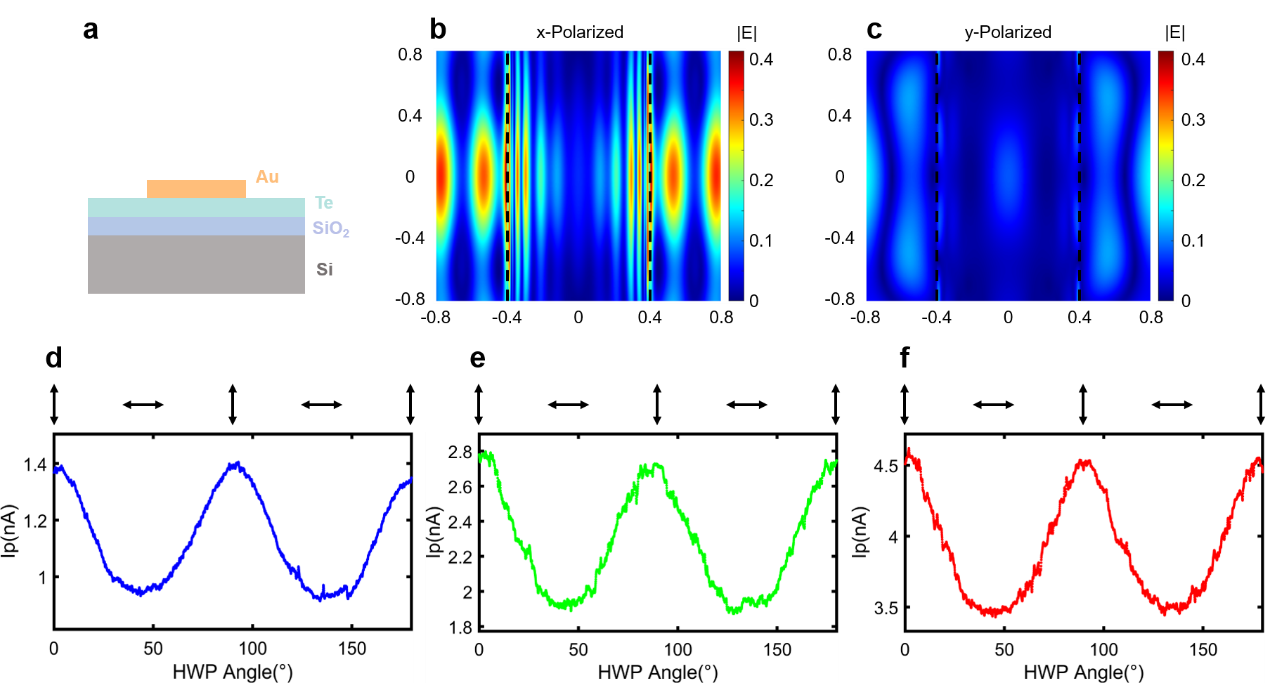


**Fig. S12 Analysis of linear polarization dependence of devices without V-grooves. a-c.** Simulation results of gold electrode scattering. Fig. S9a shows the simulated structure. Fig. S9b, c illustrate the field intensity distribution on the upper surface of Te nanosheets under x-polarized and y-polarized light excitations. The electric field intensity is markedly higher under x-polarized light due to differences in scattering efficiency. The electrode boundary is shown by the black dashed line. **d-f.** The polarization-dependent outcomes of devices without V-grooves under 405 nm, 520 nm and 638 nm wavelength. Typically, the photocurrent under y polarization is higher than that under x polarization.


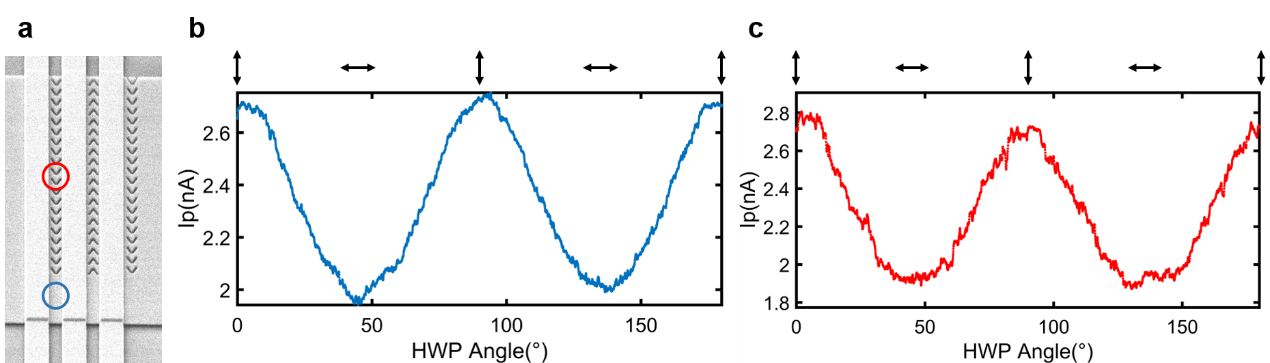


**Fig. S13 Comparison of the linear polarization dependency between devices with and without V-grooves. a.** SEM image of the tested devices, part of which was etched with V-grooves. **b.** Linear polarization dependent photoresponses of devices without V-grooves. The light spot position during measurements is indicated by the blue circle. **c.** Linear polarization dependent photoresponses of devices after etching V-grooves. The light spot was focused on the red circle locations during tests. No decisive difference is observed between them. The excitation light wavelength is 520 nm.


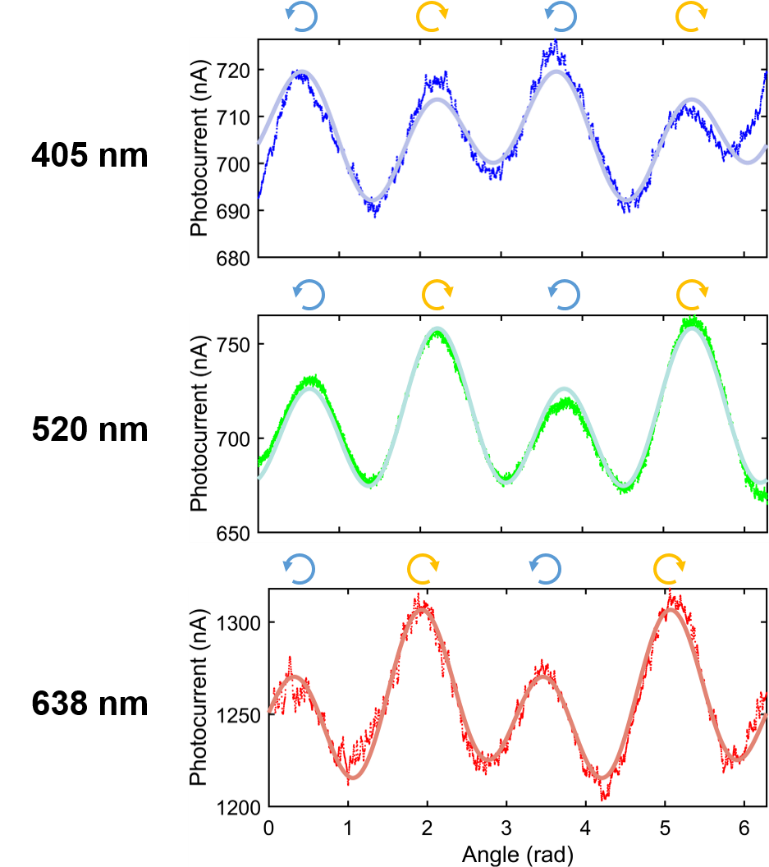


**Fig. S14 Variation of photocurrent with the rotation of the QWP under uniform illumination for a unit device.** Although CPL-sensitive responses were observed at wavelengths of 405 nm, 520 nm, and 638 nm, they do not dominate. This results in only magnitude differences in the photoresponse under LCP and RCP light excitation, with no directional distinction.


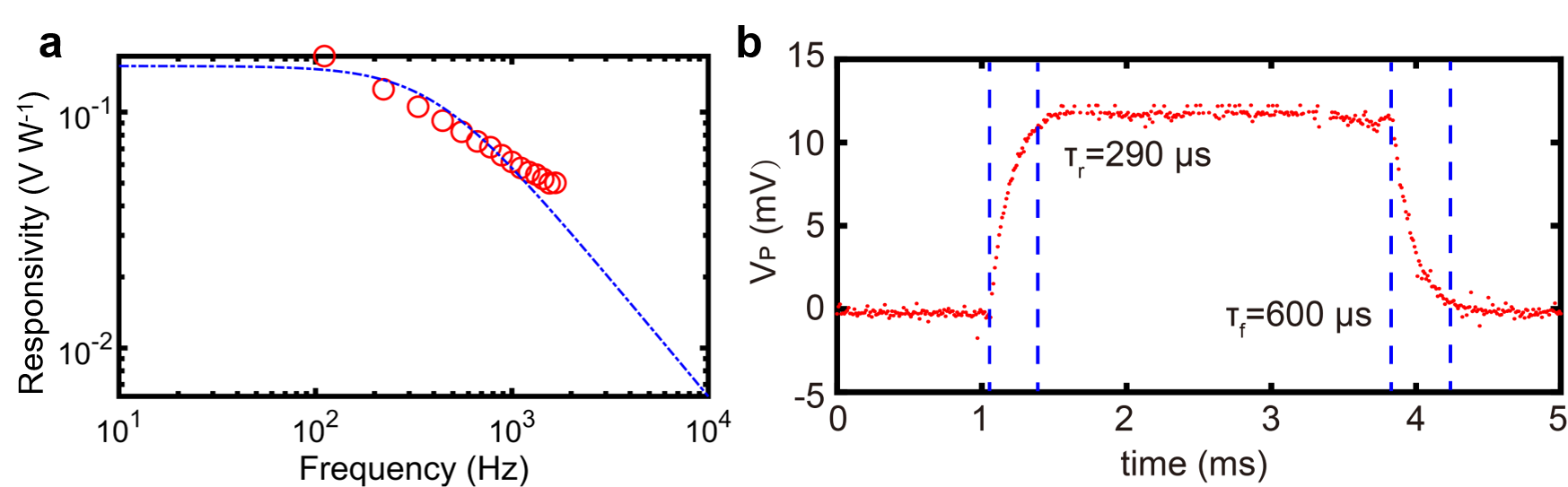


**Fig. S15 The bandwidth and rise/fall time of a device. a.** the responsivity as a function of frequency. The red circles represent the measured results, while the blue dashed line indicates the fitted curve, yielding a -3dB cutoff bandwidth of approximately 688Hz. **b.** the rise and fall times of the device's switching.


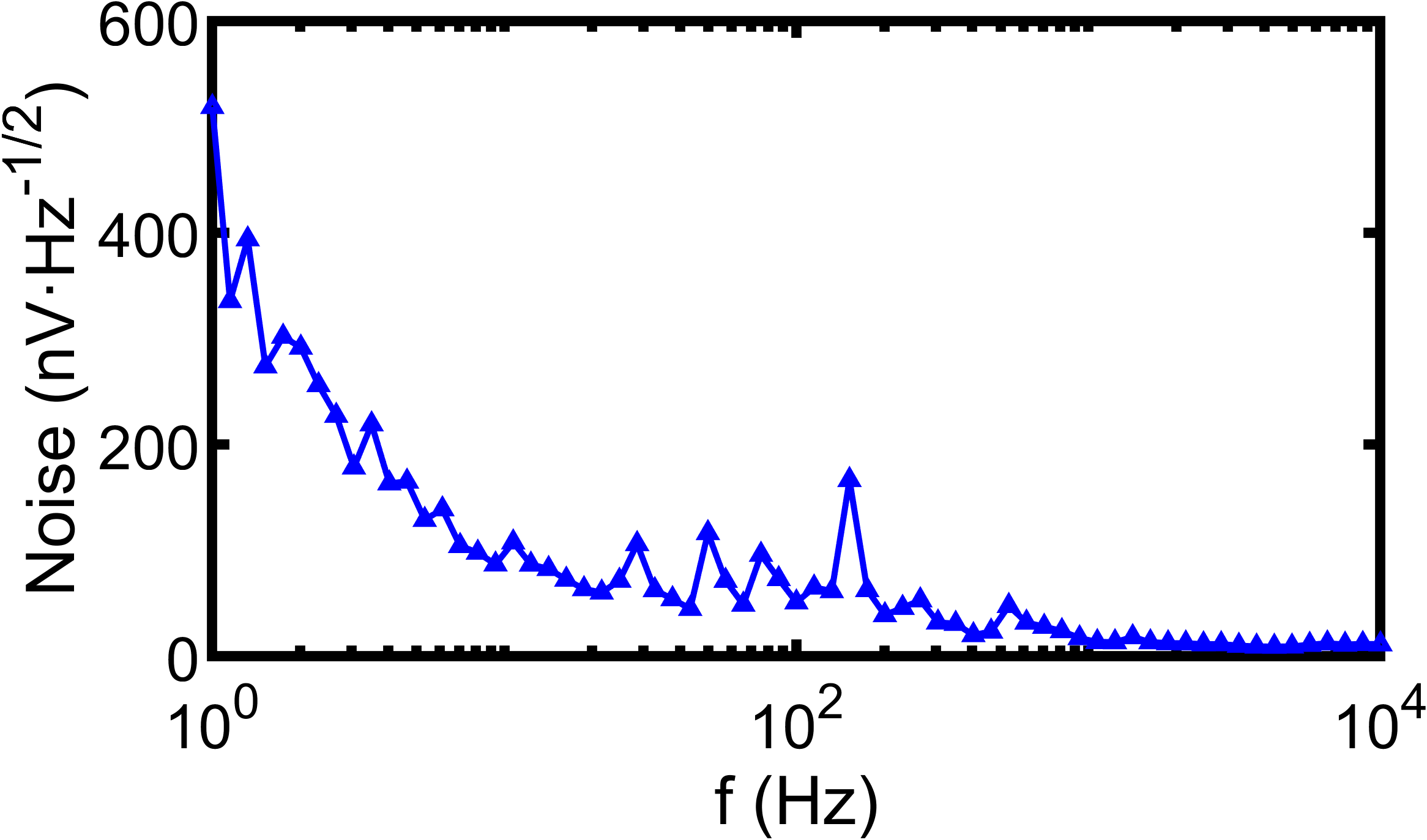


**Fig. S16 The noise spectrum of a device measured in dark conditions.**


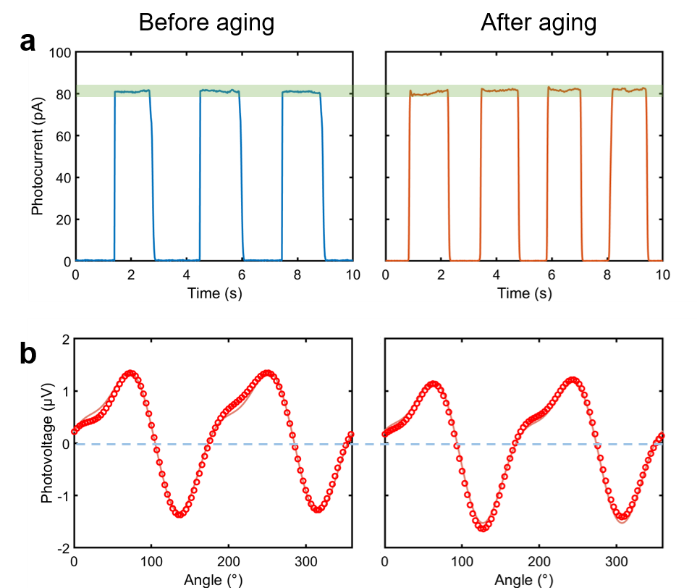


**Fig. S17 Atmospheric stability of the device.** **a.** The photoresponse of the device under 520 nm y-polarized laser before (left panel) and after (right panel) 3-month exposure in atmosphere. **b.** The CPL-dependent photoresponse of the device before and after 5-day exposure in atmosphere.

**
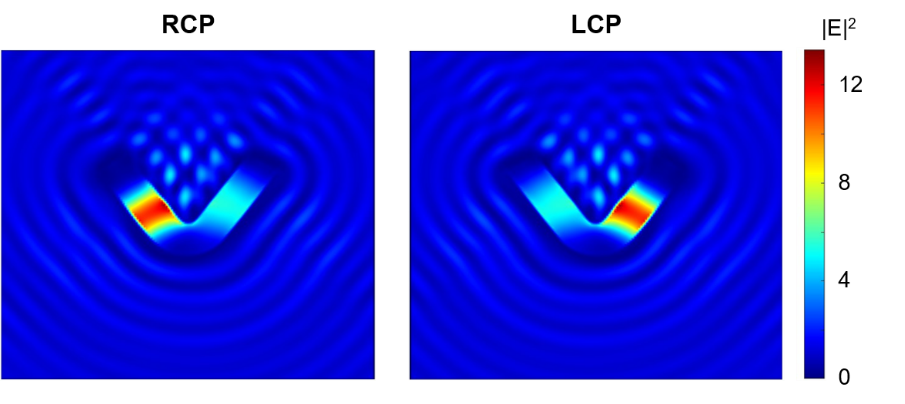
**

**Fig. S18 Chirality-sensitive near-field modes of V-shaped grooves on silicon substrates.** The thickness of the silicon layer is 500 nm, and the etching depth is 460 nm. The V-groove arm is 700 nm in length and 100 nm in width, with an angle of 70°. Under LCP and RCP excitation, the light fields are concentrated on the right and left arms of grooves respectively. It shows that the achiral-structure based CPL detection scheme can be extended to silicon and other substrate materials.


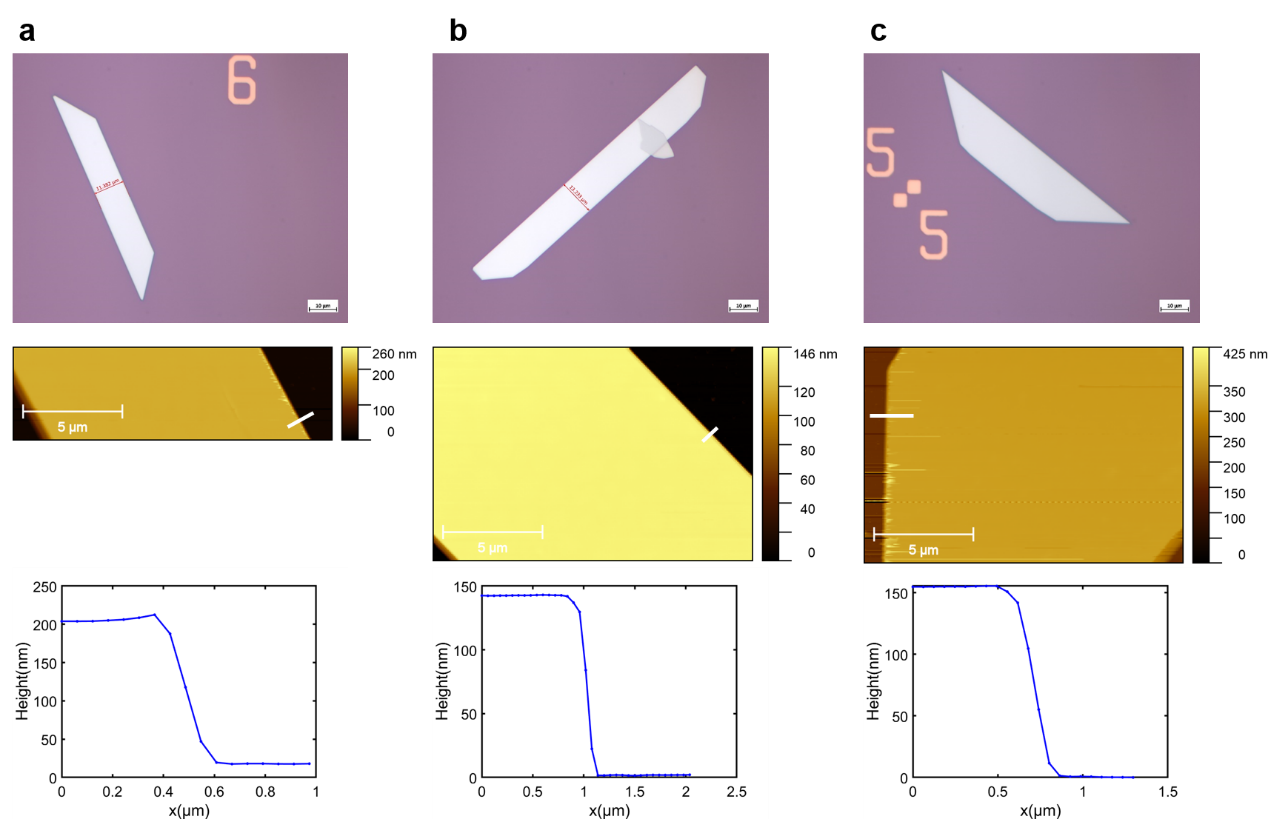


**Fig. S19 Size and thickness measurements of Te nanosheets.** The first row shows the microscopic photographs of the Te nanosheets, the second row displays the AFM images, and the third row presents the thickness of the Te nanosheets measured at the positions indicated by the white lines.


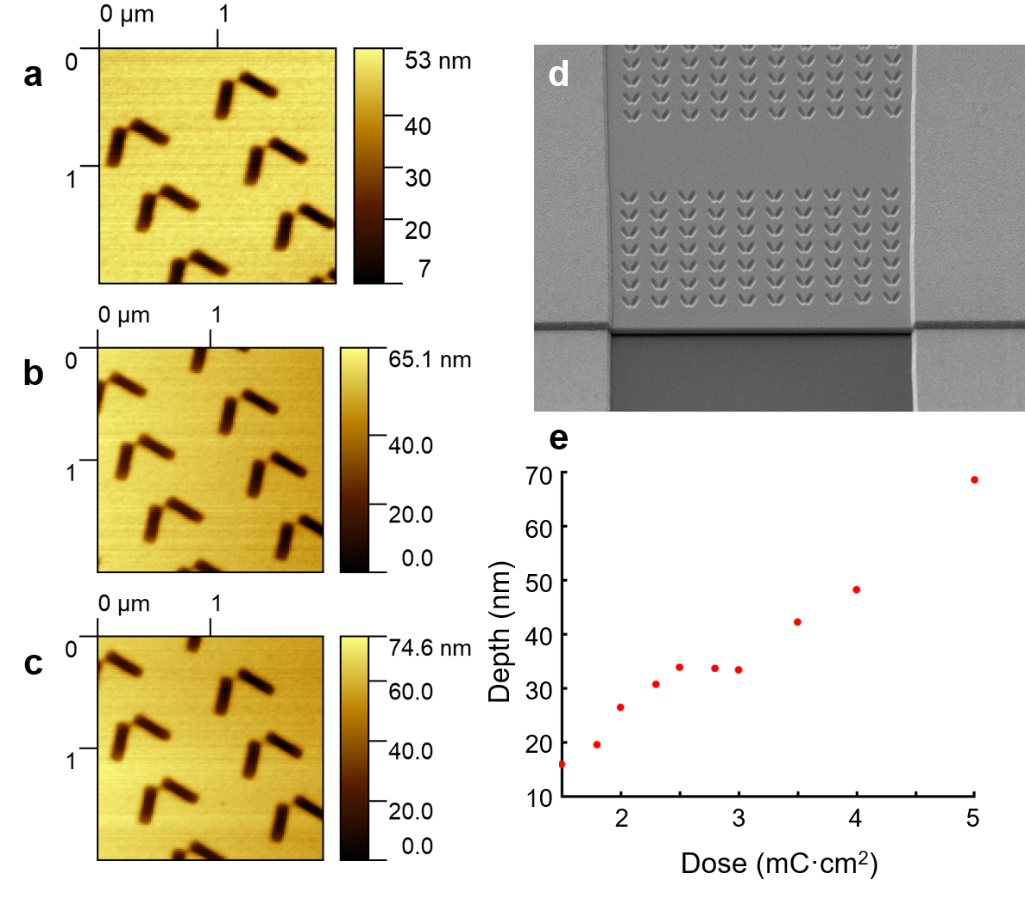


**Fig. S20 Relationship between dose and etching depth. a-c.** AFM images of V-shaped grooves etched at doses 3 μC cm^-2^, 3.5 μC cm^-2^, and 4 μC cm^-2^. **d.** SEM image of test V-grooves. **e.** V-shaped groove depth etched with different doses.

| Mechanism | Test  Wavelength | Responsivity | Discrimination  Ratio (DR) | Polarization  Ratio (PR) | Bias | EQE | Ref |
| --- | --- | --- | --- | --- | --- | --- | --- |
| Hybrid Perovskites | 395 nm | 797mA·W^-1^ | 0.1 | 1.1* | 20 V |  | ^1^ |
| Hybrid Perovskites | 395 nm | 0.28 A·W^-1^ | 1.85* | 25.4 | -0.5 V | 87.5% | ^2^ |
| Hybrid Perovskites | 405 nm | 15.7 A·W^-1^ | 0.15 | 1.16* | 2.8 V |  | ^3^ |
| Hybrid Perovskites | 500 nm | 0.14 A·W^-1^ | 0.44 | 1.56* | 0 V | 6% | ^4^ |
| Hybrid Perovskites | 520 nm | 22 μA·W^−1^@5V | 0.3@0V | 1.35* |  |  | ^5^ |
| Organic Chiral Semiconductors | 625 nm | 42.2 A·W^-1^ | 1.4 | 5.67* | 20 V |  | ^6^ |
| Organic Chiral Semiconductors | 670 nm |  | 1.2 | 4* | -1 V |  | ^7^ |
| Organic Chiral Semiconductors | 365 nm | 29.4μA·W^−1^* | 1.64* | 10* | -60 V | 0.1% | ^8^ |
| Organic Chiral Semiconductors | 375 nm |  | 0.1 | 1.1* | 5 V |  | ^9^ |
| Organic Chiral Semiconductors | 545 nm |  | 0.1 | 1.1* | 0 V | 50% | ^10^ |
| Topological Insulators | 532 nm |  | 1.74 | 14.38* | 0 V |  | ^11^ |
| Au Chiral Metasurface | 790 nm | 2.46 mA·W^-1^ | 0.38 | 1.47* | 1.5 V |  | ^12^ |
| Si Chiral Metasurface | 780 nm | 125μA·W^−1^* | 0.67* | 2* | 500 mV | 0.2%* | ^13^ |
| Au Chiral Particles | 808 nm | 0.24 A·W^-1^ | 0.55 | 1.76* | 0 V | 30.9% | ^14^ |
| Chiral Molecule -Au Particles | 808 nm |  | 0.827 | 2.41 | 0 V |  | ^15^ |
| Te Achiral Metasuface | 405 nm  520 nm  638 nm | 0.29 V·W  0.37 V·W  0.0055 V·W | 107  20  32 | -1.04  -1.23  -1.13 | 0 V |  | This work |

* indicates the parameters which are not directly given in the reference. They are calculated from the figures or other parameters.

**Table. S1** Data for representative CPL photodetectors near visible band.

1. Chen, C. *et al.* Circularly polarized light detection using chiral hybrid perovskite. *Nat Commun* **10**, 1927 (2019).

2. Ishii, A. & Miyasaka, T. Direct detection of circular polarized light in helical 1D perovskite-based photodiode. *Sci. Adv.* **6**, eabd3274 (2020).

3. Liu, T. *et al.* High Responsivity Circular Polarized Light Detectors based on Quasi Two-Dimensional Chiral Perovskite Films. *ACS Nano* **16**, 2682–2689 (2022).

4. Yao, B. *et al.* Symmetry-Broken 2D Lead–Tin Mixed Chiral Perovskite for High Asymmetry Factor Circularly Polarized Light Detection. *Nano Lett.* **23**, 1938–1945 (2023).

5. Li, D. *et al.* Chiral Lead‐Free Hybrid Perovskites for Self‐Powered Circularly Polarized Light Detection. *Angew. Chem.* **133**, 8496–8499 (2021).

6. Liu, L. *et al.* Fused-Ring Electron-Acceptor Single Crystals with Chiral 2D Supramolecular Organization for Anisotropic Chiral Optoelectronic Devices. *Adv. Mater.* **35**, 2304627 (2023).

7. Song, I. *et al.* Helical polymers for dissymmetric circularly polarized light imaging. *Nature* **617**, 92–99 (2023).

8. Yang, Y., da Costa, R. C., Fuchter, M. J. & Campbell, A. J. Circularly polarized light detection by a chiral organic semiconductor transistor. *Nat. Photonics* **7**, 634–638 (2013).

9. Kim, N. Y. *et al.* Chiroptical‐Conjugated Polymer/Chiral Small Molecule Hybrid Thin Films for Circularly Polarized Light‐Detecting Heterojunction Devices. *Adv Funct Mater* **29**, 1808668 (2019).

10. Schulz, M. *et al.* Chiral Excitonic Organic Photodiodes for Direct Detection of Circular Polarized Light. *Adv. Funct. Mater.* **29**, 1900684 (2019).

11. Sun, X. *et al.* Topological insulator metamaterial with giant circular photogalvanic effect. *Sci. Adv.* **7**, eabe5748 (2021).

12. Jiang, Q. *et al.* Ultrathin circular polarimeter based on chiral plasmonic metasurface and monolayer MoSe2. *Nanoscale* **12**, 5906–5913 (2020).

13. Hong, J. *et al.* Nonlocal metasurface for circularly polarized light detection. *Optica* **10**, 134 (2023).

14. Kim, H. *et al.* Ultrasensitive Near-Infrared Circularly Polarized Light Detection Using 3D Perovskite Embedded with Chiral Plasmonic Nanoparticles. *Adv. Sci.* **9**, 2104598 (2022).

15. Cai, J. *et al.* Polarization-sensitive optoionic membranes from chiral plasmonic nanoparticles. *Nat Nanotechnol* (2022) doi:10.1038/s41565-022-01079-3.
